# Chilblains

**Published:** 1914-08

**Authors:** 


**Chilblains.** The following application, painted on the chilblain night and morning, forms a protective skin not unlike collodion:—Acid. tannic., 8.0 gm.; Spir. rect., 16.0 c.c.; Acid. carbol., 1.5 gm.; Aquam, 30.c.c. Solve.

